# Unilateral Sensorineural Hearing Loss in Children: Etiology, Audiological Characteristics, and Treatment

**DOI:** 10.3390/children11030324

**Published:** 2024-03-09

**Authors:** Mirko Aldè, Diego Zanetti, Umberto Ambrosetti, Eleonora Monaco, Anna Maria Gasbarre, Lorenzo Pignataro, Giovanna Cantarella, Stefania Barozzi

**Affiliations:** 1 Department of Clinical Sciences and Community Health, University of Milan, 20122 Milan, Italy; mirko.alde@unimi.it (M.A.); diego.zanetti@unimi.it (D.Z.); umberto.ambrosetti@unimi.it (U.A.); eleonoramonaco@hotmail.it (E.M.); lorenzo.pignataro@unimi.it (L.P.); giovanna.cantarella@unimi.it (G.C.); 2 Audiology Unit, Department of Specialist Surgical Sciences, Fondazione IRCCS Ca’ Granda Ospedale Maggiore Policlinico, 20122 Milan, Italy; annamaria.gasbarre@policlinico.mi.it; 3 Otolaryngology Unit, Department of Specialist Surgical Sciences, Fondazione IRCCS Ca’ Granda Ospedale Maggiore Policlinico, 20122 Milan, Italy

**Keywords:** unilateral hearing loss, hearing aid, cochlear implant, children

## Abstract

The aim of this study was to evaluate audiological characteristics and parents’ opinions on hearing device use in children with unilateral sensorineural hearing loss (USNHL) who attended a tertiary-level audiologic center. The medical charts of 70 children aged 6 to 12 years with USNHL were reviewed. In 51.4% of cases, the children were diagnosed with USNHL after the age of 2 years. The main causes of USNHL were congenital cytomegalovirus infection (21.4%) and unilateral cochlear nerve hypoplasia (12.9%). The percentage of patients wearing a hearing device was 45.7% (32/70); of these, 28 (87.5%) wore a conventional hearing aid, 2 (6.3%) a CROS device, and 2 (6.3%) a cochlear implant. Regarding the choice to use a hearing device, no significant differences were found between the subcategories of hearing loss degree (*p* = 0.55) and audiometric configuration (*p* = 0.54). Most parents of children with mild-to-severe USNHL observed improved attention (90.9%), and reduced fatigue and restlessness (86.4%) using the hearing aid. These children performed significantly better on all audiological tests (speech perception in quiet and in noise conditions, and sound localization) while wearing the hearing aid (*p* < 0.001). More efforts should be made to raise awareness among professionals and parents about the negative consequences of uncorrected USNHL.

## 1. Introduction

Unilateral hearing loss (UHL) is defined as normal hearing in one ear and impaired hearing in the contralateral ear. According to the “National Workshop on mild and unilateral hearing loss”, permanent UHL is characterized by an average pure tone air conduction threshold at frequencies of 500 Hz, 1000 Hz, and 2000 Hz ≥ 20 decibels Hearing Level (dB HL) or pure tone air conduction thresholds > 25 dB HL at two or more frequencies above 2000 Hz in the impaired ear; meanwhile, the average pure tone air conduction threshold in the unaffected ear should be ≤15 dB [1]. Hearing loss (HL) can range from mild to profound, and have different audiometric configurations (flat, rising, or sloping) [2]. Specifically, single-sided deafness (SSD) refers to profound sensorineural HL or nonfunctional hearing in one ear, while the other ear maintains normal hearing [3]. The prevalence of congenital UHL is about 0.3–1 per 1000 newborns, with a remarkable increase (1.2–4.6 per 100) in newborns admitted to the neonatal intensive care unit [4]. However, it is important to underline that most children with mild HL and those with late-onset or acquired HL are not identified by universal newborn hearing screening [1,5]. Moreover, a recent study by Fitzpatrick et al. reported that nearly half of children with UHL are at risk of experiencing further hearing decline in the affected ear or developing bilateral HL [6]. The prevalence of UHL increases with age; it is estimated that 7.2% of adults in the United States of America are affected by UHL [7]. The most common causes of unilateral sensorineural hearing loss (USNHL) include premature birth, congenital or postnatal infections (especially congenital cytomegalovirus and meningitis), structural anomalies (such as cochlear nerve hypoplasia/aplasia and enlarged vestibular aqueduct), genetic underpinnings, and temporal bone trauma [6,8,9,10,11,12]; however, in many cases, the etiology remains unknown [9]. A review by Lieu showed that UHL in children has a negative impact on speech–language development, cognition, and quality of life [13]. Furthermore, Bess et al. demonstrated that children with UHL are more susceptible to listening-related fatigue and that their fatigue is akin to that of pediatric patients with bilateral HL [14]. In the past, it was traditionally believed that unilateral hearing was the minimum requirement for acceptable speech development and that, consequently, the use of hearing aids was not necessary [15]. However, since the 1980s, significant difficulties have been reported for children with UHL in the areas of localization, listening in noise, language, and academic functions [16]. In particular, Schmithorst et al. suggested that monaural hearing influences the development of brain networks associated with cross-modal sensory processing and the regulation of the default network during spoken language processing [17]. Currently, published evidence and clinical experience confirm the crucial role of the timely diagnosis and treatment of UHL in improving linguistic, cognitive, socio-emotional, and communicative development [15]. Although the treatment of USNHL is still controversial, recent comprehensive reviews have suggested that, in selected cases, cochlear implantation for children with SSD can lead to significant improvements in overall hearing outcomes [18,19]. The aim of this study was to evaluate the audiological characteristics (degree of HL, affected side, audiometric configuration, and hearing outcomes), adherence to treatment (hearing aid or cochlear implant), causes of HL, and parents’ opinions on hearing device use in children aged 6 to 12 years with USNHL who attended our tertiary-level referral audiologic center.

## 2. Materials and Methods

### 2.1. Data Collection

In the present study, all medical reports and charts of children aged 6 to 12 years with USNHL who were referred to the tertiary-level audiology center of the Fondazione IRCCS Ca’ Granda, Ospedale Maggiore Policlinico (Milan, Italy) in the period from 1 January 2021 to 31 December 2023 were reviewed.

Specifically, the study included all children who met the following criteria:

- Children 6 through 12 years of age with a confirmed diagnosis of USNHL;
- Children with at least 1 year of hearing aid or cochlear implant use in the impaired ear, or children who had never received any hearing treatment;
- Children who had undergone a comprehensive audiological evaluation, including otomicroscopy, tympanometry, reflex threshold measurements, pure tone audiometry (covering frequencies from 250 Hz to 8000 Hz), speech audiometry, Simplified Italian Matrix Test (SiIMax), and Sound Localization Test;
- Children who had undergone video head impulse test (v-HIT), high-resolution magnetic resonance imaging (MRI) of the brain, high-resolution cone beam computed tomography (CBCT) of the head, and genetic testing.

Specifically, genetic testing was performed by gene amplification (polymerase chain reaction) of the entire coding region of the 101 genes investigated, followed by sequencing analysis using next-generation sequencing (NGS) technology. The list of genes investigated was as follows: ACTG1, AIFM1, ATP6V1B1, BSND, CCDC50, CDH23, CEACAM16, CIB2, CISD2, CLDN14, CLRN1, COCH, COL11A1, COL11A2, COL2A1, COL9A1, COL9A2, CRYM, DCDC2, DFNAS (GSDME), DFNB31 (WHRN), DFNB59, DIABLO, DIAPH1, DIAPH3, EDN3, EDNRB, ESPN, ESRRB, EYA1, EYA4, FOXI1, GIPC3, GJB2, GJB3, GJB6, GPR98 (ADGRV1), GRHL2, GRXCR1, HARS, HGF, HOMER2, ILDR1, KARS, KCNE1, KCNJ10, KCNQ1, KCNQ4, KITLG, LHFPL5, LOXHD1, LRTOMT, MARVELD2, MET, MIR96, MITF, MSRB3, MYH14, MYH9, MYO15A, MYO3A, MYO6, MYO7A, NARS2, OPA1, OTOA, OTOF, PAX3, PCDH15, PDZD7, POLR1C, POLR1D, POU3F4, POU4F3, PRPS1, PTPRQ, RDX, SERPINB6, SIX1, SIX5, SLC17A8, SLC26A4 (PDS), SLC26A5, SMPX, SNAI2, SOX10, STRC, TBC1D24, TCOF1, TECTA, TJP2, TMC1, TMIE, TMPRSS3, TNC, TPRN, TRIOBP, USH1C, USH1G, USH2A, and WFS1.

Although this genetic test is very accurate, some limitations of the diagnostic method must be considered, including the inability to detect (1) other genetic diseases or genes not specifically investigated; (2) mutations located in intronic regions beyond ± 5 nucleotides from breakpoints; and (3) deletions, inversions, or duplications greater than 20 base pairs.

Family history regarding genetic predisposition to hearing impairment was also collected.

The study excluded all children with the following:

- Conductive or mixed HL;
- Congenital malformations or acquired pathologies of the outer or middle ear;
- Genetic syndromes;
- Any type of bilateral HL;
- Cognitive delay.

Degrees of HL were classified according to pure-tone averages at 500 Hz, 1000 Hz, 2000 Hz, and 4000 Hz (PTA-4). The categories of HL were distinguished into the following: mild HL, which ranged from 26 to 40 dB HL; moderate HL, from 41 to 60 dB HL; severe HL, from 61 to 80 dB HL; and profound HL, greater than 80 dB HL [20]. Regarding USNHL, the following variables were specifically investigated: age at diagnosis, affected side, HL degree, hearing changes after diagnosis, audiometric configuration, and use of a hearing device (conventional behind-the-ear [BTE] hearing aid, wireless contralateral-routing-of-signal [CROS] device, or cochlear implant). The causes of each child’s USNHL and possible associations between hearing device use and gender, degree of HL, or type of audiometric configuration were also evaluated. Additional variables investigated in children using a hearing device were as follows: age at first hearing device fitting, time elapsed between HL diagnosis and hearing device fitting, and mean daily hours of hearing device use (by analyzing the data logging of the device software). Moreover, we compared audiological test scores (PTA-4, intellection threshold, SiIMax, and Sound Localization Test) obtained without and with the hearing aid by children with mild-to-severe USNHL who regularly used a hearing aid. The “intellection threshold” was defined as the level at which the subject understood 100% of the words [21].

- *Simplified Italian Matrix Test (SiIMax)*

The SiIMax speech material included a subset of items (7 numerals, 7 nouns, and 7 adjectives) from the original 50-word base matrix of the ITAMatrix test, which means that shorter speech phrases, such as “seven white balls”, were used rather than full sentences. Specifically, each test list consisted of 14 randomized three-word sentences. Two loudspeakers were positioned at head height, respectively, 1 m in front of and behind the child sitting inside the sound booth; background noise was set at 65 dB pressure level and presented at 0° and 180° azimuth, while speech was presented at 0° azimuth and automatically adjusted according to the child’s previous response. The result of the test was reported as a score representing the signal-to-noise ratio (SNR) in dB at which the child could recognize 50% of the speech material (speech recognition threshold, SRT) [22,23].

- *Sound Localization Test*

Sound localization was tested by means of 8 loudspeakers arranged in a full circle with an angular distance of 45°; all loudspeakers were positioned at head height and labeled from number 1 to number 8. Participants were placed centrally inside the sound booth, comfortably seated on a chair, facing the loudspeaker at 0° azimuth (loudspeaker number 1), and equidistant, 1 m away, from the different loudspeakers [24]. Children were instructed not to move their head during the presentation of the auditory stimulus (a 1000 Hz warble tone presented at 40 dB sensation level for a duration of 3 s); immediately after the end of each single auditory stimulus, they had to indicate the perceived sound source by vocally reporting the number of the loudspeaker (for this purpose, they were temporarily allowed to turn their head to read the number of the loudspeaker). The level of auditory stimuli was kept fixed, as variations in intensity appeared to have no impact on sound localization [25]. The result of the test was reported as the percentage of correct responses, that is, the number of times a participant accurately identified the loudspeaker presenting the sound (n = 0–8) divided by the total number of presentations (n = 8, one per loudspeaker), multiplied by 100.

Both the SiIMax and the Sound Localization Test were performed only once after a brief trial, as these audiological examinations were explained in detail to the children by experienced audiologists before being performed.

- *Parents’ opinions on children’s use of hearing devices*

Parents of hearing-aid-wearing children with mild-to-severe USNHL were asked for their opinions on the impact of regular hearing aid use on their child’s selective attention (*“How do you perceive your child’s selective attention when he/she constantly wears the hearing aid during the day?”*), fatigue (*“How do you perceive your child’s fatigue when he/she constantly wears the hearing aid during the day?”*), restlessness (*“How do you perceive your child’s restlessness when he/she constantly wears the hearing aid during the day?”*), and overall hearing ability (*“How do you perceive your child’s hearing ability when he/she constantly wears the hearing aid during the day?”*) compared to when their child did not use the hearing aid (i.e., before hearing aid fitting, during sports activities or other special circumstances, or because the hearing aid was out of power or broken). Each question had five possible responses on a five-item Likert scale: greatly worsened = 1, worsened = 2, same = 3, improved = 4, and greatly improved = 5.

The following definitions were used [26,27,28]:

- Selective attention: child’s ability to focus on important environmental stimuli while simultaneously suppressing irrelevant or distracting information;
- Fatigue: child’s sense of tiredness, lack of energy, and feeling of exhaustion;
- Restlessness: child’s feeling of irritability and agitation;
- Hearing abilities: parental perception of the child’s hearing.

The same questions were asked to parents of children with profound USNHL who regularly used a hearing device. Children’s personal opinion of their overall hearing ability when wearing a hearing device was also analyzed (using the five-item Likert scale previously described). Finally, parents of children with mild-to-severe USNHL who did not use any hearing aids were asked the reasons for that choice, while parents of children with profound USNHL were asked why their child had never undergone cochlear implantation until then. This retrospective study was conducted in accordance with the World Medical Association Declaration of Helsinki, and received approval from the local ethics committee and parental consent.

### 2.2. Statistical Analysis

Stata 17 software (StataCorp., College Station, TX, USA, 2021) was used to perform the statistical analyses. Categorical variables were analyzed using the chi-squared test. The difference between audiological test scores without and with the hearing aid was assessed using the nonparametric Wilcoxon signed-rank sum test. A *p*-value of <0.05 was considered statistically significant.

## 3. Results

**Figure 1:**
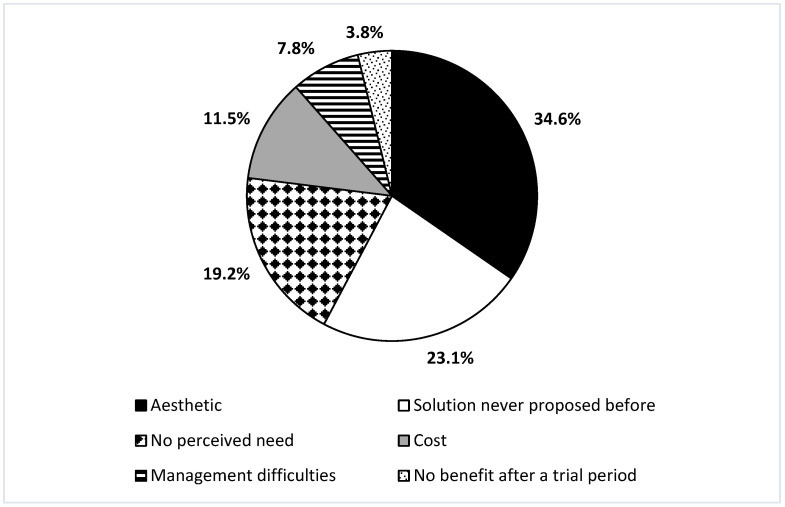
Reasons why children with mild-to-severe unilateral sensorineural hearing loss do not use a hearing aid.

A total of 70 children with USNHL, including 37 (52.9%) males and 33 (47.1%) females, met the inclusion criteria. The mean age of the study population was 8.8 ± 2.5 years, and the PTA-4 of the ear affected by USNHL was 71.1 ± 25.1 dB HL. Table 1 provides a summary of the main characteristics of USNHL observed in the study population. Among children with USNHL, 23 (32.9%) had unilaterally failed the universal neonatal hearing screening. The main causes of USNHL in the study population were congenital cytomegalovirus infection (n = 15, 21.4%) and unilateral cochlear nerve hypoplasia (n = 9, 12.9%), while, in 40% of patients (n = 28), the etiology remained unknown (Table 2). No child in the study had complete cochlear nerve aplasia, or a family history of genetic predisposition to HL. Unilateral vestibular hypofunction, detected by bedside vestibular examination and confirmed by v-HIT, was found in 14 (20%) patients; of these, 6 had profound USNHL, 5 severe USNHL, 2 moderate USNHL, and 1 mild USNHL. No significant correlation was found between the presence of unilateral vestibular hypofunction and degree of USNHL (*p* = 0.73). Regarding the choice to use a hearing device, no significant differences were found between males and females (*p* = 0.6) or between the subcategories of HL degree (*p* = 0.55) and audiometric configuration (*p* = 0.54) (Table 3). Table 4 shows the temporal characteristics of hearing treatment in children with mild-to-severe USNHL (Table 4). The mean time elapsed between HL diagnosis and hearing aid fitting was 22.0 ± 15.5 months. The mean daily hours of hearing aid use were 10.3 ± 2.8. Children with mild-to-severe USNHL who regularly used a hearing aid performed significantly better on all audiological assessments (pure tone audiometry, speech audiometry, SiIMax, and Sound Localization Test) while wearing the hearing aid (*p* < 0.001) (Table 5). Parents observed a positive impact of the hearing aid on the child’s selective attention, fatigue, restlessness, and overall hearing ability in 90.9%, 86.4%, 86.4%, and 90.9% of cases, respectively (Table 6). Among these children with mild-to-severe USNHL, 7 (31.8%) and 15 (68.2%) reported better and much better hearing with regular use of the hearing aid, respectively. The main reasons for not using a hearing aid among children with mild-to-severe USNHL were aesthetic issues (n = 9, 34.6%), followed by the fact that this solution had never been proposed before (n = 6, 23.1%) (Figure 1). Among children with profound USNHL, 2 (9.1%) had undergone cochlear implantation, 6 (27.3%) were fitted with a conventional BTE hearing aid, and 2 (9.1%) were fitted with a CROS hearing aid, while 12 (54.5%) received no hearing treatment. None of the patients included in the study used non-surgical or surgical bone conduction devices. The reasons why 90.1% (20/22) of children with profound USNHL did not undergo cochlear implantation were the following: this solution had never been proposed before (n = 10, 50%), aesthetic issues (n = 5, 25%), and parents’ concerns about surgery (n = 5, 25%). Table 7 shows the temporal characteristics of hearing treatment, the results of audiological tests without and with the hearing device, and parents’ opinions of children with profound USNHL who regularly used a hearing device (Table 7). Subjectively, both patients with a cochlear implant reported much better hearing (rated 5 out of 5 points) with regular use of the device.

## 4. Discussion

The present study investigated the causes of HL, audiological characteristics, adherence to treatment, and parents’ opinions on hearing device use in children aged 6 to 12 years with USNHL who attended a tertiary-level referral audiologic center. Interestingly, more than two-thirds of the study population had bilaterally passed the universal newborn hearing screening, and more than half of children were diagnosed with USNHL after the age of 2 years. These findings could be explained by the high proportion of cases of USNHL due to congenital cytomegalovirus infection, acquired etiologies, and other conditions associated with late-onset or progressive HL. Although universal newborn screening has some limitations [1,5], it should be emphasized that, before its introduction, the percentage of children with USNHL detected before 6 months of age was only 3% [29], compared to 35.7% in our study. In two out of five children, the etiology of USNHL remained unknown, which is consistent with what has been reported by other studies in the literature [29,30] and makes it difficult to predict the long-term hearing outcomes of these patients. Possible undetected causes of USNHL in the pediatric population might be attributable to genetic variants of unknown/uncertain significance, undiagnosed congenital cytomegalovirus infection (as most cases are asymptomatic at birth), or asymptomatic/paucisymptomatic postnatal infections [5,10,12]. This study showed that the characteristics of UNSHL are highly variable regarding age at diagnosis, affected side, degree, hearing changes over time, audiometric configuration, and use of a hearing device. Moreover, one in five children was diagnosed with unilateral vestibular hypofunction, reinforcing the need for routine vestibular and balance assessment in all pediatric patients with USNHL, as already suggested by previous studies [31,32]. Although several studies have demonstrated the negative consequences and long-term implications of uncorrected USNHL [9,13,14,15,16], less than half of our patients used a hearing device. The high percentage of non-users of hearing devices was largely attributed not only to aesthetic concerns due to social stigma, but also to parents’ lack of knowledge since this solution had never been proposed before by professionals. Although these reasons may seem surprising, they have also been reported by other studies in the literature [9,33,34,35,36], thus suggesting the need for shared decision-making processes, psychosocial supports, public health awareness campaigns, and standardized guidance from audiology services on how to manage pediatric USNHL. In our study, uncertainty about the application and acceptance of a hearing device in children with USNHL also emerged from the long time that often elapses between diagnosis and hearing device fitting, unlike what usually happens for children with bilateral SNHL [36]. In this regard, a population-based study by Fitzpatrick et al. demonstrated that more than half of children with USNHL or mild bilateral SNHL experience a considerable delay from identification to hearing aid fitting, but the reasons for this delay are often unknown [37]. As a matter of fact, parents often perceive professionals’ hesitation and lack of conviction about the potential benefits of hearing aids in children with USNHL, as well as professionals’ tendency to minimize the importance of milder HL [38]. However, only one of our patients did not use a hearing aid due to lack of benefit after an adequate trial period, confirming that most children with UNSHL who have tried a hearing device continue to use it [9,33]. The subjective benefit of wearing a hearing aid was also indirectly proven by the high mean daily hearing aid use, with all but one of our patients using the device for at least 7 h per day. Interestingly, the choice to wear a hearing device was not influenced by the degree of HL or the type of audiometric configuration. Among patients with mild-to-severe USNHL, the advantages of hearing aid use were demonstrated not only by significant improvements in sound and speech perception in quiet, speech perception in noise, and sound localization, but also by positive feedback from children and parents. In particular, most parents noticed that the use of a hearing aid improved selective attention and reduced fatigue and restlessness in their children. These findings are not surprising since children with USNHL are known to have higher levels of fatigue and irritability, and a lower performance on selective attention tests [39,40,41,42].

One of the greatest challenges is counseling families of children with profound USNHL due to the lack of robust clinical data to steer shared decision-making and identify potential benefits of unilateral cochlear implantation [18]. However, parents should be advised that children with SSD often have difficulty with sound localization and speech perception in noisy environments due to the absence of binaural auditory input [43]; in this context, unilateral cochlear implantation should be considered as a possible viable treatment aimed at restoring binaural hearing [44]. Indeed, on 19 July 2019, the Food and Drug Administration (FDA) granted approval for the MED-EL cochlear implant in children aged ≥ 5 years with SSD (maximum auditory deprivation = 10 years); Cochlear Americas received the first approval on 11 January 2022 [43]. The existing FDA guidelines for unilateral cochlear implantation stipulate that the MED-EL device requires a PTA-4 equal to or greater than 90 dB HL, while the Cochlear device requires a PTA-4 greater than 80 dB HL [43].

A recent clinical trial by Brown et al. found that pediatric patients implanted for USNHL achieved marked improvements in their ability to perceive speech in both quiet and noisy environments and to localize sounds; specifically, these children already showed head shadow and summation effects with their cochlear implant as early as 6 months after activation, whereas, by 12 months after activation, they developed a significant squelch benefit [45]. Although the age limit for cochlear implantation was set at 5 years because of the lack of rigorous clinical studies in younger children, auditory neuroplasticity declines dramatically after the age of 7 years, meaning that potential benefits for individuals with congenital SSD are confined within a short time window [46]. Therefore, it can be reasonably expected that cochlear implantation at a younger age would be beneficial in pediatric patients with profound USNHL. A recent study by Patro et al. demonstrated that children with SSD can safely undergo surgery even under the age of 5, resulting in greater improvements in speech recognition [47]. It is important to underline that some clinical conditions deserve special consideration, regardless of the patient’s age. For example, individuals with SSD due to bacterial meningitis should be implanted as soon as possible because swift ossification may obliterate the inner ear and hinder the success of the procedure [43]. Moreover, pediatric patients with SSD at risk of hearing deterioration in the contralateral ear, such as those affected by a congenital cytomegalovirus infection or an enlarged vestibular aqueduct, should promptly receive a cochlear implant not only to restore bilateral auditory input, but also to provide a lifeline in case of sudden worsening of the hearing threshold in the normal ear [5,48]. However, it should be kept in mind that candidacy for unilateral cochlear implantation requires the careful assessment of imaging, audiological testing, duration of SNHL, age, comorbid conditions, psychological support, and parental motivation [18,43]. Alternative hearing technologies available for children with SSD include re-routing devices such as CROS and bone conduction devices, but these solutions cannot provide binaural input, which means they do not enhance sound localization [9,49]. Although these devices help attenuate the head shadow effect, they can also impair speech perception when the noise comes from the impaired ear’s side [9]. In particular, CROS hearing aids may have a negative effect in complex listening scenarios if patients are unable to manage the device according to the context, which is why these types of devices are generally not recommended under the age of 5 [50]. Furthermore, it is important to remember that hearing deprivation due to SSD leads to irreversible changes in the auditory cortex, which re-routing devices are unable to prevent because they do not restore hearing in the impaired ear [9]. Given the limited amplification capacity, a conventional BTE hearing aid has few long-term benefits for children with profound USNHL. However, the auditory stimulation of the impaired ear through a well-fitted hearing aid could be useful while waiting for parents to decide on whether to have their children implanted [51,52]. In our study, among patients with profound USNHL, only the two children who had a cochlear implant presented high scores in all audiological evaluations, confirming that cochlear implantation, when not contraindicated, should be the treatment of choice for the pediatric population with SSD [18,43].

Despite the importance of these major findings, this study has several limitations. First, the study is retrospective and, because it is based on a review of medical reports and charts that were not originally intended for research data collection, some information is missing (e.g., there are no data on the mean absolute localization error, use of frequency modulation systems, detailed description of GJB2 pathogenic variants, questionnaires on children’s quality of life, children’s speech–language skills, and any speech–language therapy sessions after hearing aid fitting). Furthermore, a relatively small number of children with USNHL were evaluated in a single hospital, thus limiting the generalizability of the results. In particular, only two children had a unilateral cochlear implant. Further studies, possibly prospective, conducted across multiple centers and involving a large cohort of pediatric patients with USNHL, are necessary in order to substantiate our findings. Potential future expansions of this research might include the following: (1) an evaluation of long-term audiological and vestibular outcomes in children with USNHL; (2) an assessment of sound localization, speech perception in both quiet and noisy environments, and speech–language skills before and several months after hearing device fitting; and (3) translation and adaptation into the Italian language of some existing English questionnaires aimed at estimating children’s listening abilities in different everyday situations, such as the Children’s Home Inventory for Listening Difficulties (C.H.I.L.D.) [53].

## 5. Conclusions

The present study showed that a high percentage of patients with USNHL were diagnosed after the age of 2 years, confirming the importance of performing regular surveillance of developmental milestones and auditory skills in all children, regardless of newborn hearing screening outcome. Although all children underwent extensive diagnostic evaluations, in many cases, the etiology of USNHL was idiopathic. The management of children with USNHL should include the accurate and timely diagnosis of HL, appropriate selection of amplification devices, verification of the fitted devices, and long-term audiological follow-up. Cochlear implantation should be considered a viable treatment option for children with SSD, especially those at risk of hearing deterioration in the contralateral ear. More efforts should be made to raise awareness among professionals and parents about the negative consequences of uncorrected USNHL. Rigorous and updated national and international guidelines for the management of pediatric UHL are a priority.

## Figures and Tables

**Table 1:** Characteristics of unilateral sensorineural hearing loss in the study population.

Variables	Patients, N (%)
**Age at diagnosis**	
<6 months	25 (35.7)
6–24 months	9 (12.9)
25–60 months	20 (28.6)
>60 months	16 (22.9)
**Side**	
Right	37 (52.9)
Left	33 (47.1)
**Degree**	
Mild	8 (11.4)
Moderate	14 (20.0)
Severe	26 (37.1)
Profound	22 (31.4)
**Hearing changes after diagnosis**	
No changes	58 (82.9)
Improvement	2 (2.9)
Deterioration	10 (14.3)
**Audiometric configuration**	
Rising	6 (8.6)
Flat	40 (57.1)
Sloping	24 (34.3)
**Hearing device**	
None	38 (54.3)
Hearing aid	30 (42.9)
Cochlear implant	2 (2.9)
**TOTAL**	**70 (100.0)**

**Table 2:** Causes of unilateral sensorineural hearing loss in the study population.

Etiology	Patients, N (%)
**(1) Infectious**	**21 (30.0)**
Congenital cytomegalovirus	15 (21.4)
Epstein–Barr virus	4 (5.7)
Bacterial meningitis	2 (2.9)
**(2) Otologic**	**15 (21.4)**
Cochlear nerve hypoplasia	9 (12.9)
Enlarged vestibular aqueduct	4 (5.7)
Superior semicircular canal dehiscence	2 (2.9)
**(3) Other causes**	**6 (8.6)**
Prematurity	4 (5.7)
Pathogenic mutations in GJB2 gene	2 (2.9)
**(4) Unknown**	**28 (40.0)**
**TOTAL**	**70 (100.0)**

**Table 3:** Demographic and audiological characteristics of patients with unilateral sensorineural hearing loss using a hearing device.

Variables	Total (N)	Patients with a Hearing Device, N (%)	*p*-Value
**Gender**			
Male	37	18 (48.6)	0.6
Female	33	14 (42.4)	
**Degree of hearing loss**			
Mild	8	2 (25.0)	0.55
Moderate	14	6 (42.9)	
Severe	26	14 (53.8)	
Profound	22	10 (45.5)	
**Audiometric configuration**			
Rising	6	2 (33.3)	0.54
Flat	40	17 (42.5)	
Sloping	24	13 (54.2)	
**TOTAL**	**70**	**32 (45.7)**	

**Table 4:** Temporal characteristics of hearing treatment in children with mild-to-severe unilateral sensorineural hearing loss.

Variables	Patients, N (%)
**Age at first hearing aid fitting**	
<6 months	0 (0.0)
6–24 months	2 (9.1)
25–60 months	10 (45.5)
>60 months	10 (45.5)
**Time elapsed between hearing loss diagnosis and hearing aid fitting**	
<6 months	2 (9.1)
6–12 months	6 (27.3)
13–36 months	11 (50.0)
>36 months	3 (13.6)
**Daily hours of hearing aid use**	
<6 h	1 (4.5)
7–10 h	9 (40.9)
>10 h	12 (54.5)
**TOTAL**	**22 (100.0)**

**Table 5:** Hearing tests performed without and with the hearing aid by children with mild-to-severe unilateral sensorineural hearing loss who regularly used a hearing aid.

	Without Hearing Aid (Mean ± sd)	With Hearing Aid (Mean ± sd)	*p*-Value
**PTA-4 (dB)**	60.4 ± 10.1	30.2 ± 6.8	<0.001
**Intellection threshold (dB)**	80.5 ± 16.2	40.5 ± 7.9	<0.001
**Simplified Italian Matrix Test (dB)**	−1.8 ± 1.0	−4.3 ± 0.9	<0.001
**Sound Localization Test (%)**	51.7 ± 15.1	90.9 ± 8.8	<0.001

PTA = pure-tone averages. Intellection threshold = level at which the subject understands 100% of the words.

**Table 6:** Parents’ opinions on the impact of the hearing aid on selective attention, fatigue, restlessness, and overall hearing ability of their children with mild-to-severe unilateral sensorineural hearing loss.

Variables	Parents’ Opinions, N (%)
**Selective attention [1–5 points]**	
Greatly worsened [1]	0 (0.0)
Worsened [2]	0 (0.0)
Same [3]	2 (9.1)
Improved [4]	7 (31.8)
Greatly improved [5]	13 (59.1)
**Fatigue [1–5]**	
Greatly worsened [1]	0 (0.0)
Worsened [2]	0 (0.0)
Same [3]	3 (13.6)
Improved [4]	12 (54.5)
Greatly improved [5]	7 (31.8)
**Restlessness [1–5]**	
Greatly worsened [1]	0 (0.0)
Worsened [2]	0 (0.0)
Same [3]	3 (13.6)
Improved [4]	10 (45.5)
Greatly improved [5]	9 (40.9)
**Overall hearing ability [1–5]**	
Greatly worsened [1]	0 (0.0)
Worsened [2]	0 (0.0)
Same [3]	2 (9.1)
Improved [4]	8 (36.4)
Greatly improved [5]	12 (54.5)
**TOTAL**	**22 (100.0)**

**Table 7:** Temporal characteristics of hearing treatment, audiological test results, and parents’ opinions of children with profound unilateral sensorineural hearing loss using a hearing device.

Variables	Cochlear Implant		Conventional Hearing Aid						CROS	
	P1	P2	P3	P4	P5	P6	P7	P8	P9	P10
**Gender**	F	M	F	F	M	M	M	M	F	M
**Age** (years)	7	6	10	8	11	11	11	6	7	8
**Side**	R	L	L	R	R	R	L	R	L	R
**Age at first hearing device fitting** (months)	60	48	84	60	60	84	70	36	60	72
**Time elapsed between hearing loss diagnosis and hearing device fitting** (months)	57	45	20	54	52	14	30	33	57	69
**Daily hours of hearing device use** (hours)	14	12	10	12	10	10	12	14	8	8
**PTA-4 without hearing device** (dB)	102.5	107.5	95.5	90	100	96.3	95	88.8	110	105
**PTA-4 with hearing device** (dB)	25	20	67.5	65	75	70	65	63.8	/	/
**Maximum speech intelligibility without hearing device** (%)	0	0	20	20	0	10	20	30	0	0
**Maximum speech intelligibility with hearing device** (%)	100	100	50	40	30	40	50	50	/	/
**Simplified Italian matrix test without hearing device** (dB)	−1.2	−0.8	−2.4	−1.8	−2.2	−3	−2.2	−1.8	−1.2	−1
**Simplified Italian matrix test with hearing device** (dB)	−5.2	−5	−3.4	−3.0	−3.2	−3.8	−3.6	−3.2	−2.0	−1.8
**Sound Localization Test without hearing device (%)**	50	37.5	50	50	37.5	37.5	37.5	50	37.5	37.5
**Sound Localization Test with hearing device (%)**	100	100	62.5	62.5	62.5	62.5	62.5	87.5	37.5	37.5
**Parents’ opinions [1–5]**										
Selective attention [1–5]	5	5	4	4	4	3	4	4	3	4
Fatigue [1–5]	5	5	4	3	4	4	3	4	4	4
Restlessness [1–5]	5	4	4	4	3	4	4	4	3	3
Overall hearing ability [1–5]	5	5	4	4	3	4	3	4	3	3

F = female. L = left. M = male. P = patient. PTA = pure-tone average. R = right.

## Data Availability

The data presented in this study are available upon request from the corresponding author.
